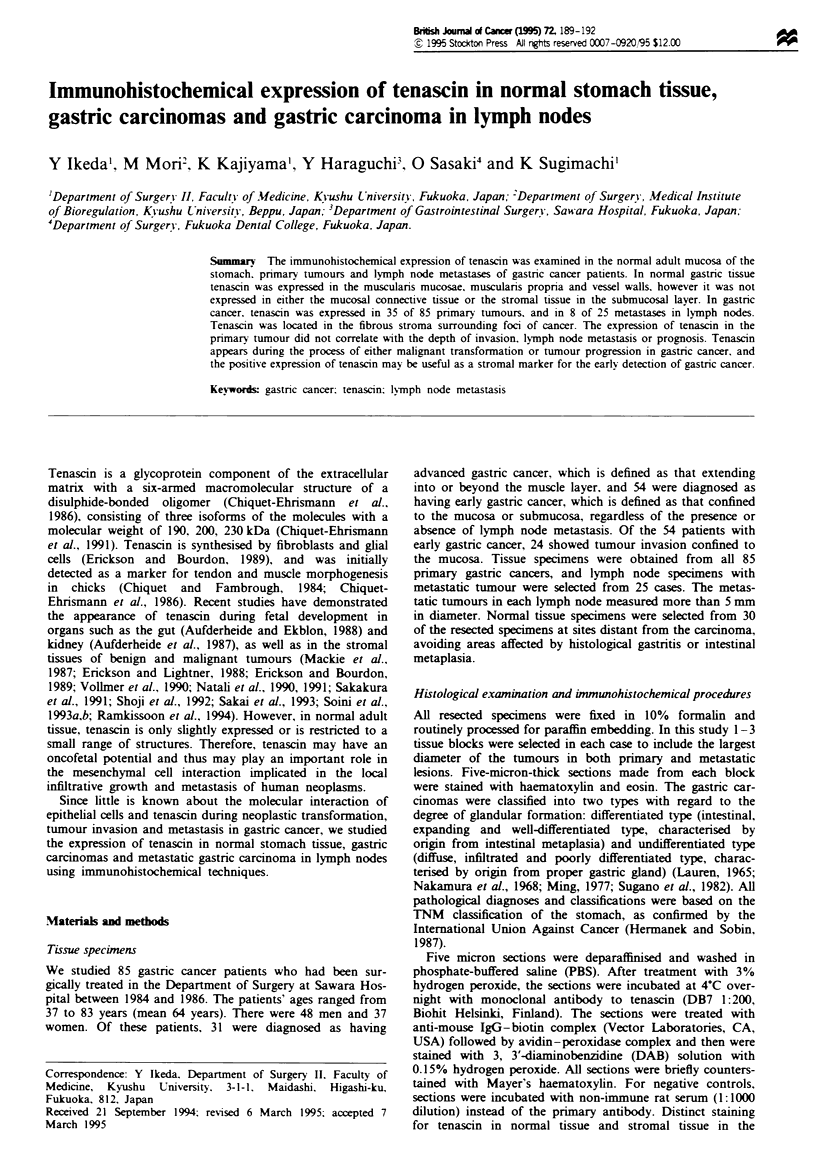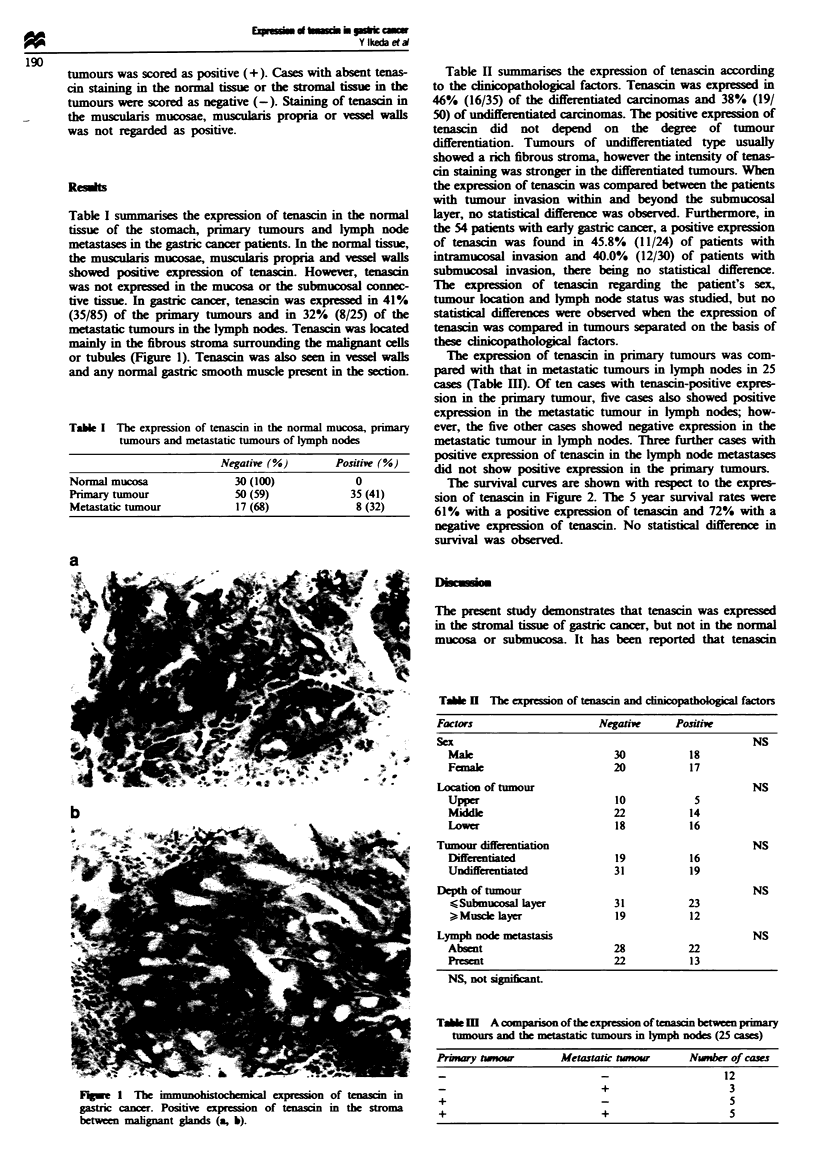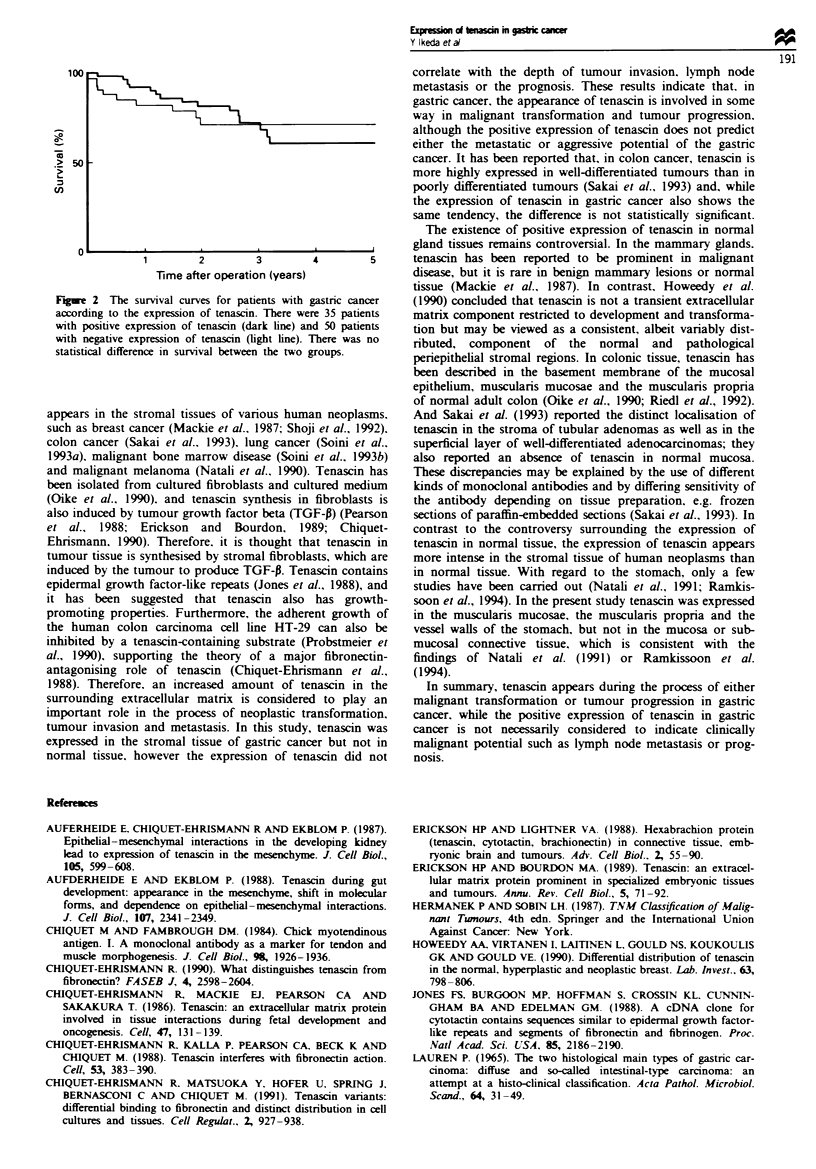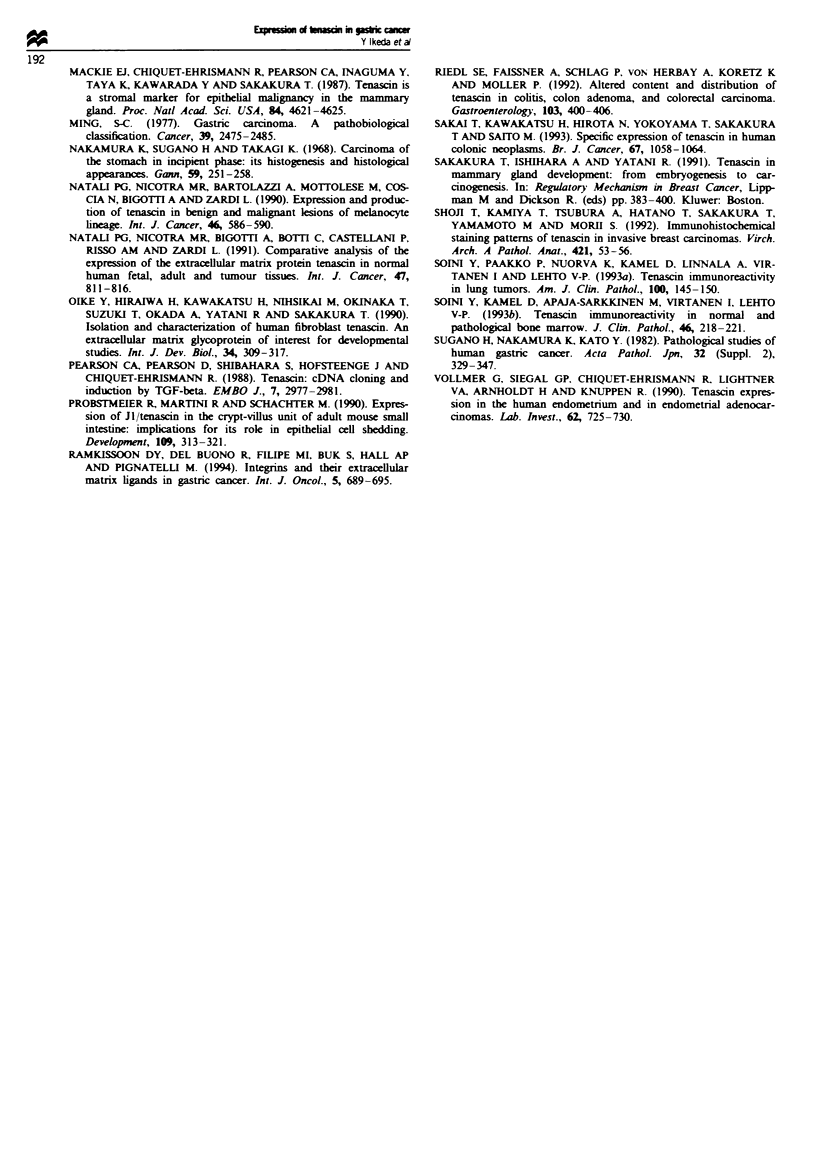# Immunohistochemical expression of tenascin in normal stomach tissue, gastric carcinomas and gastric carcinoma in lymph nodes.

**DOI:** 10.1038/bjc.1995.301

**Published:** 1995-07

**Authors:** Y. Ikeda, M. Mori, K. Kajiyama, Y. Haraguchi, O. Sasaki, K. Sugimachi

**Affiliations:** Department of Surgery II, Faculty of Medicine, Kyushu University, Fukuoka, Japan.

## Abstract

**Images:**


British Journal of Cancer (1995) 72, 189-192

© 1995 Stockton Press All rights reserved 0007-0920/95 $12.00

### Immunohistochemical expression of tenascin in normal stomach tissue, gastric carcinomas and gastric carcinoma in lymph nodes

Y Ikeda[1], M Mori[2], K Kajiyama[1], Y Haraguchi[3], O Sasaki[4] and K Sugimachi[1]

[1]*Department of Surgery II, Faculty of Medicine, Kyushu University, Fukuoka, Japan;* [2]*Department of Surgery, Medical Institute of Bioregulation, Kyushu University, Beppu, Japan;* [3]*Department of Gastrointestinal Surgery, Sawara Hospital, Fukuoka, Japan;* [4]*Department of Surgery, Fukuoka Dental College, Fukuoka, Japan.*

**Summary** The immunohistochemical expression of tenascin was examined in the normal adult mucosa of the stomach, primary tumours and lymph node metastases of gastric cancer patients. In normal gastric tissue tenascin was expressed in the muscularis mucosae, muscularis propria and vessel walls, however it was not expressed in either the mucosal connective tissue or the stromal tissue in the submucosal layer. In gastric cancer, tenascin was expressed in 35 of 85 primary tumours, and in 8 of 25 metastases in lymph nodes. Tenascin was located in the fibrous stroma surrounding foci of cancer. The expression of tenascin in the primary tumour did not correlate with the depth of invasion, lymph node metastasis or prognosis. Tenascin appears during the process of either malignant transformation or tumour progression in gastric cancer, and the positive expression of tenascin may be useful as a stromal marker for the early detection of gastric cancer.

**Keywords:** gastric cancer; tenascin; lymph node metastasis

Tenascin is a glycoprotein component of the extracellular matrix with a six-armed macromolecular structure of a disulphide-bonded oligomer (Chiquet-Ehrismann *et al.*, 1986), consisting of three isoforms of the molecules with a molecular weight of 190, 200, 230 kDa (Chiquet-Ehrismann *et al.*, 1991). Tenascin is synthesised by fibroblasts and glial cells (Erickson and Bourdon, 1989), and was initially detected as a marker for tendon and muscle morphogenesis in chicks (Chiquet and Fambrough, 1984; Chiquet-Ehrismann *et al.*, 1986). Recent studies have demonstrated the appearance of tenascin during fetal development in organs such as the gut (Aufderheide and Ekblon, 1988) and kidney (Aufderheide *et al.*, 1987), as well as in the stromal tissues of benign and malignant tumours (Mackie *et al.*, 1987; Erickson and Lightner, 1988; Erickson and Bourdon, 1989; Vollmer *et al.*, 1990; Natali *et al.*, 1990, 1991; Sakakura *et al.*, 1991; Shoji *et al.*, 1992; Sakai *et al.*, 1993; Soini *et al.*, 1993*a*,*b*; Ramkissoon *et al.*, 1994). However, in normal adult tissue, tenascin is only slightly expressed or is restricted to a small range of structures. Therefore, tenascin may have an oncofetal potential and thus may play an important role in the mesenchymal cell interaction implicated in the local infiltrative growth and metastasis of human neoplasms.

Since little is known about the molecular interaction of epithelial cells and tenascin during neoplastic transformation, tumour invasion and metastasis in gastric cancer, we studied the expression of tenascin in normal stomach tissue, gastric carcinomas and metastatic gastric carcinoma in lymph nodes using immunohistochemical techniques.

### Materials and methods

### *Tissue specimens*

We studied 85 gastric cancer patients who had been surgically treated in the Department of Surgery at Sawara Hospital between 1984 and 1986. The patients' ages ranged from 37 to 83 years (mean 64 years). There were 48 men and 37 women. Of these patients, 31 were diagnosed as having advanced gastric cancer, which is defined as that extending into or beyond the muscle layer, and 54 were diagnosed as having early gastric cancer, which is defined as that confined to the mucosa or submucosa, regardless of the presence or absence of lymph node metastasis. Of the 54 patients with early gastric cancer, 24 showed tumour invasion confined to the mucosa. Tissue specimens were obtained from all 85 primary gastric cancers, and lymph node specimens with metastatic tumour were selected from 25 cases. The metastatic tumours in each lymph node measured more than 5 mm in diameter. Normal tissue specimens were selected from 30 of the resected specimens at sites distant from the carcinoma, avoiding areas affected by histological gastritis or intestinal metaplasia.

### *Histological examination and immunohistochemical procedures*

All resected specimens were fixed in 10% formalin and routinely processed for paraffin embedding. In this study 1-3 tissue blocks were selected in each case to include the largest diameter of the tumours in both primary and metastatic lesions. Five-micron-thick sections made from each block were stained with haematoxylin and eosin. The gastric carcinomas were classified into two types with regard to the degree of glandular formation: differentiated type (intestinal, expanding and well-differentiated type, characterised by origin from intestinal metaplasia) and undifferentiated type (diffuse, infiltrated and poorly differentiated type, characterised by origin from proper gastric gland) (Lauren, 1965; Nakamura *et al.*, 1968; Ming, 1977; Sugano *et al.*, 1982). All pathological diagnoses and classifications were based on the TNM classification of the stomach, as confirmed by the International Union Against Cancer (Hermanek and Sobin, 1987).

Five micron sections were deparaffinised and washed in phosphate-buffered saline (PBS). After treatment with 3% hydrogen peroxide, the sections were incubated at 4°C overnight with monoclonal antibody to tenascin (DB7 1:200, Biohit Helsinki, Finland). The sections were treated with anti-mouse IgG-biotin complex (Vector Laboratories, CA, USA) followed by avidin-peroxidase complex and then were stained with 3, 3'-diaminobenzidine (DAB) solution with 0.15% hydrogen peroxide. All sections were briefly counterstained with Mayer's haematoxylin. For negative controls, sections were incubated with non-immune rat serum (1:1000 dilution) instead of the primary antibody. Distinct staining for tenascin in normal tissue and stromal tissue in the

Correspondence: Y Ikeda, Department of Surgery II, Faculty of Medicine, Kyushu University, 3-1-1, Maidashi, Higashi-ku, Fukuoka, 812, Japan
Received 21 September 1994; revised 6 March 1995; accepted 7 March 1995

tumours was scored as positive (+). Cases with absent tenascin staining in the normal tissue or the stromal tissue in the tumours were scored as negative (−). Staining of tenascin in the muscularis mucosae, muscularis propria or vessel walls was not regarded as positive.

### Results

Table I summarises the expression of tenascin in the normal tissue of the stomach, primary tumours and lymph node metastases in the gastric cancer patients. In the normal tissue, the muscularis mucosae, muscularis propria and vessel walls showed positive expression of tenascin. However, tenascin was not expressed in the mucosa or the submucosal connective tissue. In gastric cancer, tenascin was expressed in 41% (35/85) of the primary tumours and in 32% (8/25) of the metastatic tumours in the lymph nodes. Tenascin was located mainly in the fibrous stroma surrounding the malignant cells or tubules (Figure 1). Tenascin was also seen in vessel walls and any normal gastric smooth muscle present in the section.

**Table I** The expression of tenascin in the normal mucosa, primary tumours and metastatic tumours of lymph nodes

| | *Negative (%)* | *Positive (%)* |
|---|---|---|
| Normal mucosa | 30 (100) | 0 |
| Primary tumour | 50 (59) | 35 (41) |
| Metastatic tumour | 17 (68) | 8 (32) |

**Figure 1** The immunohistochemical expression of tenascin in gastric cancer. Positive expression of tenascin in the stroma between malignant glands (**a**, **b**).

Table II summarises the expression of tenascin according to the clinicopathological factors. Tenascin was expressed in 46% (16/35) of the differentiated carcinomas and 38% (19/50) of undifferentiated carcinomas. The positive expression of tenascin did not depend on the degree of tumour differentiation. Tumours of undifferentiated type usually showed a rich fibrous stroma, however the intensity of tenascin staining was stronger in the differentiated tumours. When the expression of tenascin was compared between the patients with tumour invasion within and beyond the submucosal layer, no statistical difference was observed. Furthermore, in the 54 patients with early gastric cancer, a positive expression of tenascin was found in 45.8% (11/24) of patients with intramucosal invasion and 40.0% (12/30) of patients with submucosal invasion, there being no statistical difference. The expression of tenascin regarding the patient's sex, tumour location and lymph node status was studied, but no statistical differences were observed when the expression of tenascin was compared in tumours separated on the basis of these clinicopathological factors.

The expression of tenascin in primary tumours was compared with that in metastatic tumours in lymph nodes in 25 cases (Table III). Of ten cases with tenascin-positive expression in the primary tumour, five cases also showed positive expression in the metastatic tumour in lymph nodes; however, the five other cases showed negative expression in the metastatic tumour in lymph nodes. Three further cases with positive expression of tenascin in the lymph node metastases did not show positive expression in the primary tumours.

The survival curves are shown with respect to the expression of tenascin in Figure 2. The 5 year survival rates were 61% with a positive expression of tenascin and 72% with a negative expression of tenascin. No statistical difference in survival was observed.

### Discussion

The present study demonstrates that tenascin was expressed in the stromal tissue of gastric cancer, but not in the normal mucosa or submucosa. It has been reported that tenascin

**Table II** The expression of tenascin and clinicopathological factors

| *Factors* | *Negative* | *Positive* | |
|---|---|---|---|
| Sex | | | NS |
| Male | 30 | 18 | |
| Female | 20 | 17 | |
| Location of tumour | | | NS |
| Upper | 10 | 5 | |
| Middle | 22 | 14 | |
| Lower | 18 | 16 | |
| Tumour differentiation | | | NS |
| Differentiated | 19 | 16 | |
| Undifferentiated | 31 | 19 | |
| Depth of tumour | | | NS |
| $\leqslant$Submucosal layer | 31 | 23 | |
| $\geqslant$Muscle layer | 19 | 12 | |
| Lymph node metastasis | | | NS |
| Absent | 28 | 22 | |
| Present | 22 | 13 | |

NS, not significant.

**Table III** A comparison of the expression of tenascin between primary tumours and the metastatic tumours in lymph nodes (25 cases)

| *Primary tumour* | *Metastatic tumour* | *Number of cases* |
|---|---|---|
| − | − | 12 |
| − | + | 3 |
| + | − | 5 |
| + | + | 5 |

**Figure 2** The survival curves for patients with gastric cancer according to the expression of tenascin. There were 35 patients with positive expression of tenascin (dark line) and 50 patients with negative expression of tenascin (light line). There was no statistical difference in survival between the two groups.

appears in the stromal tissues of various human neoplasms, such as breast cancer (Mackie *et al.*, 1987; Shoji *et al.*, 1992), colon cancer (Sakai *et al.*, 1993), lung cancer (Soini *et al.*, 1993*a*), malignant bone marrow disease (Soini *et al.*, 1993*b*) and malignant melanoma (Natali *et al.*, 1990). Tenascin has been isolated from cultured fibroblasts and cultured medium (Oike *et al.*, 1990), and tenascin synthesis in fibroblasts is also induced by tumour growth factor beta (TGF-$\beta$) (Pearson *et al.*, 1988; Erickson and Bourdon, 1989; Chiquet-Ehrismann, 1990). Therefore, it is thought that tenascin in tumour tissue is synthesised by stromal fibroblasts, which are induced by the tumour to produce TGF-$\beta$. Tenascin contains epidermal growth factor-like repeats (Jones *et al.*, 1988), and it has been suggested that tenascin also has growth-promoting properties. Furthermore, the adherent growth of the human colon carcinoma cell line HT-29 can also be inhibited by a tenascin-containing substrate (Probstmeier *et al.*, 1990), supporting the theory of a major fibronectin-antagonising role of tenascin (Chiquet-Ehrismann *et al.*, 1988). Therefore, an increased amount of tenascin in the surrounding extracellular matrix is considered to play an important role in the process of neoplastic transformation, tumour invasion and metastasis. In this study, tenascin was expressed in the stromal tissue of gastric cancer but not in normal tissue, however the expression of tenascin did not correlate with the depth of tumour invasion, lymph node metastasis or the prognosis. These results indicate that, in gastric cancer, the appearance of tenascin is involved in some way in malignant transformation and tumour progression, although the positive expression of tenascin does not predict either the metastatic or aggressive potential of the gastric cancer. It has been reported that, in colon cancer, tenascin is more highly expressed in well-differentiated tumours than in poorly differentiated tumours (Sakai *et al.*, 1993) and, while the expression of tenascin in gastric cancer also shows the same tendency, the difference is not statistically significant.

The existence of positive expression of tenascin in normal gland tissues remains controversial. In the mammary glands, tenascin has been reported to be prominent in malignant disease, but it is rare in benign mammary lesions or normal tissue (Mackie *et al.*, 1987). In contrast, Howeedy *et al.* (1990) concluded that tenascin is not a transient extracellular matrix component restricted to development and transformation but may be viewed as a consistent, albeit variably distributed, component of the normal and pathological periepithelial stromal regions. In colonic tissue, tenascin has been described in the basement membrane of the mucosal epithelium, muscularis mucosae and the muscularis propria of normal adult colon (Oike *et al.*, 1990; Riedl *et al.*, 1992). And Sakai *et al.* (1993) reported the distinct localisation of tenascin in the stroma of tubular adenomas as well as in the superficial layer of well-differentiated adenocarcinomas; they also reported an absence of tenascin in normal mucosa. These discrepancies may be explained by the use of different kinds of monoclonal antibodies and by differing sensitivity of the antibody depending on tissue preparation, e.g. frozen sections of paraffin-embedded sections (Sakai *et al.*, 1993). In contrast to the controversy surrounding the expression of tenascin in normal tissue, the expression of tenascin appears more intense in the stromal tissue of human neoplasms than in normal tissue. With regard to the stomach, only a few studies have been carried out (Natali *et al.*, 1991; Ramkissoon *et al.*, 1994). In the present study tenascin was expressed in the muscularis mucosae, the muscularis propria and the vessel walls of the stomach, but not in the mucosa or submucosal connective tissue, which is consistent with the findings of Natali *et al.* (1991) or Ramkissoon *et al.* (1994).

In summary, tenascin appears during the process of either malignant transformation or tumour progression in gastric cancer, while the positive expression of tenascin in gastric cancer is not necessarily considered to indicate clinically malignant potential such as lymph node metastasis or prognosis.

### References

AUFERHEIDE E, CHIQUET-EHRISMANN R AND EKBLOM P. (1987). Epithelial-mesenchymal interactions in the developing kidney lead to expression of tenascin in the mesenchyme. *J. Cell Biol.*, **105**, 599-608.

AUFDERHEIDE E AND EKBLOM P. (1988). Tenascin during gut development: appearance in the mesenchyme, shift in molecular forms, and dependence on epithelial-mesenchymal interactions. *J. Cell Biol.*, **107**, 2341-2349.

CHIQUET M AND FAMBROUGH DM. (1984). Chick myotendinous antigen. I. A monoclonal antibody as a marker for tendon and muscle morphogenesis. *J. Cell Biol.*, **98**, 1926-1936.

CHIQUET-EHRISMANN R. (1990). What distinguishes tenascin from fibronectin? *FASEB J*, **4**, 2598-2604.

CHIQUET-EHRISMANN R, MACKIE EJ, PEARSON CA AND SAKAKURA T. (1986). Tenascin: an extracellular matrix protein involved in tissue interactions during fetal development and oncogenesis. *Cell*, **47**, 131-139.

CHIQUET-EHRISMANN R, KALLA P, PEARSON CA, BECK K AND CHIQUET M. (1988). Tenascin interferes with fibronectin action. *Cell*, **53**, 383-390.

CHIQUET-EHRISMANN R, MATSUOKA Y, HOFER U, SPRING J, BERNASCONI C AND CHIQUET M. (1991). Tenascin variants: differential binding to fibronectin and distinct distribution in cell cultures and tissues. *Cell Regulat.*, **2**, 927-938.

ERICKSON HP AND LIGHTNER VA. (1988). Hexabrachion protein (tenascin, cytotactin, brachionectin) in connective tissue, embryonic brain and tumours. *Adv. Cell Biol.*, **2**, 55-90.

ERICKSON HP AND BOURDON MA. (1989). Tenascin: an extracellular matrix protein prominent in specialized embryonic tissues and tumours. *Annu. Rev. Cell Biol.*, **5**, 71-92.

HERMANEK P AND SOBIN LH. (1987). *TNM Classification of Malignant Tumours*, 4th edn. Springer and the International Union Against Cancer: New York.

HOWEEDY AA, VIRTANEN I, LAITINEN L, GOULD NS, KOUKOULIS GK AND GOULD VE. (1990). Differential distribution of tenascin in the normal, hyperplastic and neoplastic breast. *Lab. Invest.*, **63**, 798-806.

JONES FS, BURGOON MP, HOFFMAN S, CROSSIN KL, CUNNINGHAM BA AND EDELMAN GM. (1988). A cDNA clone for cytotactin contains sequences similar to epidermal growth factor-like repeats and segments of fibronectin and fibrinogen. *Proc. Natl Acad. Sci. USA*, **85**, 2186-2190.

LAUREN P. (1965). The two histological main types of gastric carcinoma: diffuse and so-called intestinal-type carcinoma: an attempt at a histo-clinical classification. *Acta Pathol. Microbiol. Scand.*, **64**, 31-49.

MACKIE EJ, CHIQUET-EHRISMANN R, PEARSON CA, INAGUMA Y, TAYA K, KAWARADA Y AND SAKAKURA T. (1987). Tenascin is a stromal marker for epithelial malignancy in the mammary gland. *Proc. Natl Acad. Sci. USA*, **84**, 4621-4625.

MING, S-C. (1977). Gastric carcinoma. A pathobiological classification. *Cancer*, **39**, 2475-2485.

NAKAMURA K, SUGANO H AND TAKAGI K. (1968). Carcinoma of the stomach in incipient phase: its histogenesis and histological appearances. *Gann*, **59**, 251-258.

NATALI PG, NICOTRA MR, BARTOLAZZI A, MOTTOLESE M, COSCIA N, BIGOTTI A AND ZARDI L. (1990). Expression and production of tenascin in benign and malignant lesions of melanocyte lineage. *Int. J. Cancer*, **46**, 586-590.

NATALI PG, NICOTRA MR, BIGOTTI A, BOTTI C, CASTELLANI P, RISSO AM AND ZARDI L. (1991). Comparative analysis of the expression of the extracellular matrix protein tenascin in normal human fetal, adult and tumour tissues. *Int. J. Cancer*, **47**, 811-816.

OIKE Y, HIRAIWA H, KAWAKATSU H, NIHSIKAI M, OKINAKA T, SUZUKI T, OKADA A, YATANI R AND SAKAKURA T. (1990). Isolation and characterization of human fibroblast tenascin. An extracellular matrix glycoprotein of interest for developmental studies. *Int. J. Dev. Biol.*, **34**, 309-317.

PEARSON CA, PEARSON D, SHIBAHARA S, HOFSTEENGE J AND CHIQUET-EHRISMANN R. (1988). Tenascin: cDNA cloning and induction by TGF-beta. *EMBO J.*, **7**, 2977-2981.

PROBSTMEIER R, MARTINI R AND SCHACHTER M. (1990). Expression of J1/tenascin in the crypt-villus unit of adult mouse small intestine: implications for its role in epithelial cell shedding. *Development*, **109**, 313-321.

RAMKISSOON DY, DEL BUONO R, FILIPE MI, BUK S, HALL AP AND PIGNATELLI M. (1994). Integrins and their extracellular matrix ligands in gastric cancer. *Int. J. Oncol.*, **5**, 689-695.

RIEDL SE, FAISSNER A, SCHLAG P, VON HERBAY A, KORETZ K AND MOLLER P. (1992). Altered content and distribution of tenascin in colitis, colon adenoma, and colorectal carcinoma. *Gastroenterology*, **103**, 400-406.

SAKAI T, KAWAKATSU H, HIROTA N, YOKOYAMA T, SAKAKURA T AND SAITO M. (1993). Specific expression of tenascin in human colonic neoplasms. *Br. J. Cancer*, **67**, 1058-1064.

SAKAKURA T, ISHIHARA A AND YATANI R. (1991). Tenascin in mammary gland development: from embryogenesis to carcinogenesis. In: *Regulatory Mechanism in Breast Cancer*, Lippman M and Dickson R. (eds) pp. 383-400. Kluwer: Boston.

SHOJI T, KAMIYA T, TSUBURA A, HATANO T, SAKAKURA T, YAMAMOTO M AND MORII S. (1992). Immunohistochemical staining patterns of tenascin in invasive breast carcinomas. *Virch. Arch. A Pathol. Anat.*, **421**, 53-56.

SOINI Y, PAAKKO P, NUORVA K, KAMEL D, LINNALA A, VIRTANEN I AND LEHTO V-P. (1993*a*). Tenascin immunoreactivity in lung tumors. *Am. J. Clin. Pathol.*, **100**, 145-150.

SOINI Y, KAMEL D, APAJA-SARKKINEN M, VIRTANEN I, LEHTO V-P. (1993*b*). Tenascin immunoreactivity in normal and pathological bone marrow. *J. Clin. Pathol.*, **46**, 218-221.

SUGANO H, NAKAMURA K, KATO Y. (1982). Pathological studies of human gastric cancer. *Acta Pathol. Jpn*, **32** (Suppl. 2), 329-347.

VOLLMER G, SIEGAL GP, CHIQUET-EHRISMANN R, LIGHTNER VA, ARNHOLDT H AND KNUPPEN R. (1990). Tenascin expression in the human endometrium and in endometrial adenocarcinomas. *Lab. Invest.*, **62**, 725-730.